# Integrative genomic and clinical analysis of carbapenem-resistant and susceptible *Klebsiella pneumoniae* isolates from a tertiary hospital in Wuhan

**DOI:** 10.3389/fcimb.2026.1881548

**Published:** 2026-08-12

**Authors:** Mengling Liu, Zhiyong Wu, Jieyu Mao, Shuo Zhang, Sheng Xu, Jinwen Min, Wenjing Yu, Haizhou Liu, Yangbo Hu, Xiaojun Wu

**Affiliations:** 1 Department of Pulmonary and Critical Care Medicine, Renmin Hospital of Wuhan University, Wuhan University, Wuhan, China; 2 Key Laboratory of Virology and Biosafety, Wuhan Institute of Virology, Chinese Academy of Sciences, Wuhan, China; 3 University of Chinese Academy of Sciences, Beijing, China

**Keywords:** carbapenem-resistant *Klebsiella pneumoniae*, carbapenem-susceptible *Klebsiella pneumoniae*, clinical risk factors, comparative genomics, pan-genome

## Abstract

**Objectives:**

Carbapenem-resistant *Klebsiella pneumoniae* (CRKP) has emerged as a major global health threat due to its resistance to carbapenems. As the mechanisms underlying carbapenem resistance in CRKP remain incompletely understood, we seek to provide important insights into the clinical and genomic characteristics of CRKP.

**Methods:**

A total of 40 CRKP and 42 carbapenem-susceptible *Klebsiella pneumoniae* (CSKP) isolates were collected from a tertiary hospital in Wuhan, China. Integrating 2,460 publicly available *Klebsiella pneumoniae* genomes, we conducted a comprehensive analysis of clinical risk factors, resistance and virulence gene profiles, plasmid replicon composition, core genome SNP-based phylogeny, pan-genome structure, and genome-wide single-nucleotide variants and insertions/deletions (SNVs/InDels) variation.

**Results:**

Clinical analysis identified a Charlson comorbidity index > 3 and respiratory tract infections as independent risk factors for CRKP infection. Genomic comparisons revealed that CRKP strains harbored more resistance and virulence genes than CSKP strains, with ST11 being the dominant CRKP clone that frequently carried the *blaKPC-2* gene. Phylogenetic analysis incorporating public genomes revealed regional differences in sequence type and carbapenemase genotype distributions. Pan-genome analysis demonstrated open pan-genome characteristics in both groups, with cloud genes representing the largest gene category. Comparative genomic analyses revealed distinct SNV and InDel distribution patterns between CRKP and CSKP isolates, while GO annotation of the prioritized variant-associated genes showed broad functional representation across cellular and metabolic processes, transport and responses to stimuli, membrane-associated components, and binding and catalytic functions.

**Conclusions:**

CCI >3 and respiratory tract infection were independently associated with CRKP infection. ST11-*blaKPC-2* was the predominant epidemic lineage and CRKP isolates carried a greater burden of resistance and virulence determinants than CSKP isolates. Phylogenetic, pan-genomic, and comparative genomic analyses revealed substantial genomic diversity and distinct genomic variation patterns between CRKP and CSKP. These findings provide insights into the clinical risk profile, clonal dynamics, and genomic evolution of CRKP, informing future surveillance and infection control strategies.

## 1 Introduction

*Klebsiella pneumoniae* (*K. pneumoniae*) is a clinically important opportunistic pathogen responsible for severe community- and hospital-acquired infections (Biondo, 2023). However, the extensive use of antibiotics has accelerated the emergence and dissemination of carbapenem-resistant *K. pneumoniae* (CRKP), which represents a major threat to the efficacy of last-line β-lactam antibiotics (Wang et al., 2022; Shi et al., 2024; Wang et al., 2024). CRKP infections are associated with prolonged hospitalization, increased healthcare burden, and higher mortality, posing a serious challenge to global public health (Wang et al., 2022).

CRKP has become a global concern, with substantial geographic variation in dominant clones and resistance mechanisms. ST258/ST512 carrying *blaKPC* are widely distributed in North America and Europe, whereas ST11 carrying *blaKPC-2* has emerged as the predominant CRKP lineage in China and other Asian regions (Peirano et al., 2017; Shi et al., 2026). High-risk clones such as ST147 and ST307 have also been increasingly reported worldwide, highlighting the dynamic evolution and dissemination of CRKP populations (Peirano et al., 2020). Although carbapenemase production, particularly KPC-2 in China, remains the major mechanism of carbapenem resistance, other mechanisms including porin alterations, efflux pump overexpression, and chromosomal mutations may also contribute to resistance (Liao et al., 2020; Wang et al., 2024). In addition, virulence-associated factors such as capsular polysaccharides, adhesins, lipopolysaccharides, and siderophore systems contribute to the pathogenic potential of *K. pneumoniae* (Lin et al., 2024). The co-carriage of *rmpA/rmpA2* and siderophore genes (*iucA/B/C/D, iutA, iroB/C/D/N*) is often linked to pLVPK-like virulence plasmids (Chen et al., 2004; Gu et al., 2018). The emergence of strains carrying both resistance and virulence determinants further complicates clinical management (Chen et al., 2024).

Despite extensive epidemiological and genomic studies, the differences between CRKP and carbapenem-susceptible *K. pneumoniae* (CSKP) remain incompletely understood, particularly regarding the interplay between resistance determinants and genomic variation. This study aimed to systematically investigate the clinical and genomic characteristics of CRKP compared with CSKP isolates collected from a tertiary hospital in Wuhan, China, providing insights into the epidemiology and evolution of CRKP and informing future surveillance and infection control strategies.

## 2 Methods

### 2.1 Patients and bacterial isolates

Non-duplicate *K. pneumoniae* isolates were consecutively collected from various clinical specimens of hospitalized patients in different departments of the Renmin Hospital of Wuhan University between March and August, 2024. The main inclusion criteria were as follows (Biondo, 2023): *K. pneumoniae* was the predominant species in the culture or was isolated from a sterile site, and (Shi et al., 2024) symptomatic infection at the *K. pneumoniae* culture site. For respiratory tract isolates, infection was defined based on compatible clinical manifestations and/or radiological findings consistent with pneumonia. Isolates considered colonization, without clinical evidence of active infection, were excluded. Strains from different specimens of the same patient or from specimens of the same patient collected at different times were considered duplicate strains, and only the first strain was included in subsequent studies. All isolates were identified using the BD Phoenix 100 system (Becton, Dickinson and Company, MD, USA) and confirmed using matrix-assisted laser desorption ionization time-of-flight mass spectrometry (Bruker Daltonik, Bremen, Germany). Demographic and clinical data of the corresponding patients were retrieved from the hospital’s electronic medical record system. This study was approved by the research ethics committee of Renmin Hospital of Wuhan University under the ethics approval code WDRY2024-K190.

### 2.2 Antimicrobial susceptibility testing

A total of 82 K*. pneumoniae* strains were analyzed, comprising 40 CRKP and 42 CSKP isolates. Antimicrobial susceptibility testing was conducted using the Kirby-Bauer disc diffusion method, and the minimum inhibitory concentrations were determined using automated broth microdilution with the BD Phoenix 100 system. The susceptibility breakpoints were interpreted according to the Clinical and Laboratory Standards Institute (CLSI) guideline M100, 30th edition (2020) (Clinical and Laboratory Standards Institute, 2020). Antibiotics were categorized into different classes according to their mechanisms of action for comparative analysis of genotype–phenotype relationships. CRKP was defined as *K. pneumoniae* resistant to at least one carbapenem agent, including imipenem, meropenem, or ertapenem.

### 2.3 Bioinformatics analysis

#### 2.3.1 Whole-genome sequencing, assembly, and annotation

Genomic DNA from the *K. pneumoniae* isolates was extracted using the TIANamp Bacteria DNA Kit (Tiangen Biotech, Beijing, China), and whole-genome sequencing was performed on an Illumina NovaSeq 6000 platform. Raw reads were assessed before filtering using FastQC (Andrews, 2014), and isolate-level raw-read quality metrics, including FastQC module status, GC content, sequence length and total sequence counts, are provided in **Supplementary Table 1**. Raw paired-end reads were quality-filtered using fastp v0.23.4 (Chen et al., 2018) with default parameters. Under the default fastp settings, bases with Phred quality scores below Q15 were treated as unqualified bases, reads containing more than 40% unqualified bases were discarded, and reads shorter than 15 bp after trimming were removed. The default fastp settings were applied uniformly across all isolates to maintain a reproducible preprocessing workflow and to preserve sufficient read depth for bacterial draft-genome assembly. Adapter trimming and read filtering were performed automatically during fastp preprocessing. The filtered reads were assembled *de novo* using SPAdes v3.15.5 (Bankevich et al., 2012) in isolate mode with a maximum memory allocation of 500 GB. Assembly quality was assessed for each isolate by calculating total assembly length, GC content, number of contigs, N50 and related contiguity metrics. The complete *de novo* assembly quality metrics for all isolates are provided in **Supplementary Table 2**.

Resistance and virulence genes were identified using RGI v5.1.1 (Alcock et al., 2023) and ABRicate v1.0.1 (Maguire et al., 2025), respectively. Antimicrobial resistance genes were detected against CARD v3.0.9 (Alcock et al., 2023), retaining only Perfect and Strict RGI hits. Virulence genes were screened against the virulence factor database (VFDB, release dated 4 November 2023) (Chen et al., 2005), with hits retained at ≥80% nucleotide identity and ≥80% coverage.

Multilocus sequence typing (MLST) was performed using MLST v2.23.0 (Urwin and Maiden, 2003). Reference-based single-nucleotide variation (SNV) and insertions/deletion (InDel) calling was performed using Bowtie2 v2.5.4 (Langmead and Salzberg, 2012) and Snippy v4.6.0 (Dedrick et al., 2023), with NC_016845.1 as the reference chromosome. NC_016845.1 represents the complete circular chromosome of *K. pneumoniae* subsp. *pneumoniae* HS11286. This reference was selected because it is a complete RefSeq chromosome with stable genomic coordinates and curated gene annotations, facilitating consistent read mapping, variant calling and gene-level functional annotation.Snippy was run with default parameters. Briefly, reads were mapped to NC_016845.1, and SNVs and InDels were called using Snippy’s default filtering criteria, including a minimum read depth of 10, minimum base quality of 13, minimum mapping quality of 60 and minimum alternate allele proportion of 0.9. Genome coverage and sequencing depth were also assessed from the read-mapping results. The resulting high-confidence SNV and InDel calls were used for downstream comparative genomic analyses. Because this was a reference-based analysis, variant interpretation was restricted to regions alignable to NC_016845.1 and was not considered a comprehensive assessment of accessory or non-reference genomic regions. The results were visualized using the R packages *circlize* v0.4.16 (Gu et al., 2014) and *ComplexHeatmap* v2.22.0 (Gu, 2022).

#### 2.3.2 Pan-genome and evolutionary analysis

A total of 2,460 publicly available *K. pneumoniae* genomes were retrieved from the NCBI Genome database on 31 October 2024. Only RefSeq-designated assemblies assigned to *K. pneumoniae* and annotated at the “complete genome” or “chromosome” assembly level were retained. Assemblies labelled as atypical, excluded from RefSeq, metagenome-derived, lacking species-level assignment, or available only as contig- or scaffold-level drafts were removed before downstream comparative and phylogenetic analyses. Resistance and virulence genes in public genomes were identified using the same pipeline as that for local isolates, and MLST was conducted. For genome annotation, Prokka v1.14.6 (Seemann, 2014) was used to annotate both the in-house assembled scaffolds and public genome sequences. Pan-genome analysis was performed using Roary v3.13.0 (Page et al., 2015) to generate a core gene set. Core single-nucleotide polymorphisms (SNPs) were extracted from the core gene alignment, retaining only variable sites present in at least 1% of the strains. For phylogenetic analysis, IQ-TREE v2.2.2.7 (Nguyen et al., 2015) was used to construct a maximum likelihood phylogenetic tree based on core SNPs, using the parameters -bb 1000 -bnni -MFP. The resulting phylogenetic tree was visualized using the R packages *ggtree* (v3.14.0) (Xu et al., 2022) and *ggtreeExtra* (v1.16.0) (Xu et al., 2021). Based on the previous scaffold assemblies, plasmids in *K. pneumoniae* were identified using PlasmidFinder v2.1.6 (Carattoli and Hasman, 2020), and the results were visualized with *ggplot2* (Coleman et al., 2023) in combination with the detected antimicrobial resistance and virulence genes.

#### 2.3.3 Differential pan-genome analysis and functional annotation of SNV/InDel-associated genes between CRKP and CSKP

To identify accessory genes differentially distributed between CRKP and CSKP isolates, Prokka annotations from both groups were jointly analyzed using Roary within a unified pan-genome framework. Roary was run with the parameter -cd 100, and core genes were excluded from subsequent accessory-gene association analysis. For each non-core gene cluster, prevalence was compared between CRKP and CSKP isolates using two-sided Fisher’s exact tests, with *P* values adjusted using the Benjamini–Hochberg false-discovery-rate procedure. CRKP-associated accessory genes were defined as those with FDR < 0.05, a CRKP-CSKP prevalence difference ≥0.20, and a prevalence ≥50% among CRKP isolates. To extend the analysis from individual genes to broader functional patterns, the resulting CRKP-associated gene set was used as the foreground for COG, GO, and KEGG enrichment analyses based on eggNOG-derived annotations, with all tested accessory genes serving as the background. Over-representation was assessed using one-sided Fisher’s exact tests followed by Benjamini-Hochberg correction. Terms with FDR < 0.05 and at least three foreground genes were considered significantly enriched. Fold enrichment was calculated relative to the corresponding annotation-specific background, and KEGG pathway identifiers were mapped to official pathway names before visualization.

To further characterize the genomic regions highlighted by the SNV and InDel analyses, a Random Forest classifier with 100 trees was used to distinguish CRKP from CSKP isolates based on gene-level SNV/InDel features. The dataset was initially divided into training and test sets at a ratio of 70:30 using a fixed random seed of 42. No importance-based feature selection was performed before model fitting. Model robustness was further evaluated using repeated stratified five-fold cross-validation with 20 repeats, generating 100 validation evaluations. Performance was assessed using ROC-AUC. Feature importance was calculated for each cross-validation model. Mean importance, standard deviation, feature rank and top-100 retention frequency were used to evaluate feature stability. The retained genes were functionally annotated by integrating results from comprehensive antibiotic resistance database (CARD), VFDB, BacMet2 (Pal et al., 2014), and eggNOG (Cantalapiedra et al., 2021). Resistance-associated genes were identified using CARD v3.0.9. Virulence-associated genes were screened using ABRicate against the VFDB (release dated 4 November 2023). Metal- or biocide-resistance-associated genes were identified using the experimentally confirmed BacMet2 database file *BacMet2_EXP_database.fasta* from the official BacMet2 distribution, with a directory timestamp of 2017-12-06. Broader functional annotation was performed using eggNOG v5.0.2, including complementary classification of putative adaptive or fitness-related functions and Gene Ontology annotation summarized across the biological process, cellular component, and molecular function domains.

### 2.4 Statistical analysis

Continuous variables were analyzed based on their distribution characteristics. Normally distributed data were expressed as mean ± standard deviation, while non-normally distributed data were expressed as median (interquartile range, IQR). Categorical variables were denoted as frequencies (percentages). For antimicrobial resistance phenotype, resistance gene, and virulence gene analyses, percentages were calculated as the number of isolates exhibiting the corresponding phenotype or carrying the corresponding resistance or virulence-associated gene divided by the total number of isolates in the respective group. Categorical data were analyzed using Pearson’s chi-square, Fisher’s exact, or McNemar’s tests, as appropriate. Continuous variables were compared using a two independent samples t-test or Mann–Whitney U test, as appropriate. Univariate analyses were conducted to identify the factors associated with CRKP infection. Variables with P < 0.05 in the univariate analysis were selected as candidate variables for multivariable analysis. Multicollinearity among candidate variables was assessed using variance inflation factors (VIFs), and VIF > 5 was considered indicative of significant multicollinearity. As all variables showed acceptable VIF values (<5), they were subsequently entered into a forward likelihood ratio (Forward LR) binary logistic regression model. Model calibration was evaluated using the Hosmer–Lemeshow goodness-of-fit test, and model discrimination was assessed using receiver operating characteristic (ROC) curve analysis. Odds ratios (OR), 95% confidence intervals (CI), and *P* values were calculated. Variables with *P* < 0.05 and OR > 1 were considered independent risk factors. Statistical analyses were performed using the SPSS software (version 26.0; IBM Corp., Armonk, NY, USA). Statistical significance was set at *P* < 0.05 (two-sided).

## 3 Results

### 3.1 Clinical and demographic characteristics

A total of 82 patients with *K. pneumoniae* infection were enrolled, with a median age of 62.0 years (IQR, 55.0–74.0), including 63 males. Based on carbapenem susceptibility, the patients were categorized into CRKP (n = 40) and CSKP (n = 42) groups. Patients with CRKP infection had a higher mortality rate (27.5% vs. 7.1%, *P* = 0.014) and longer hospital stay (length of stay [days]: 30, 27.5% vs. 16.7%, *P* = 0.236) than those with CSKP infection. The clinical and demographic characteristics of the *K. pneumoniae*-infected patients are summarized in **Supplementary Table 3**, **S4**.

Nine variables with *P* < 0.05 in the univariate analysis were further evaluated for multicollinearity. All variables showed acceptable collinearity statistics (VIFs ranging from 1.108 to 3.306), indicating no significant multicollinearity. Subsequently, these variables were entered into a forward likelihood ratio multivariable logistic regression model. Respiratory tract infection (OR = 9.096, 95% CI: 3.055–27.082, *P* < 0.001) and Charlson Comorbidity Index > 3 (OR = 5.489, 95% CI: 1.611–18.696, *P* = 0.006) were identified as independent risk factors for CRKP infection (**Table 1**). The Hosmer–Lemeshow goodness-of-fit test demonstrated adequate calibration of the model (*P* = 0.466). Receiver operating characteristic (ROC) curve analysis showed acceptable discriminative ability, with an area under the curve (AUC) of 0.782 (95% CI: 0.682–0.882, *P* < 0.001).

**Table 1:** Univariate and multivariate analysis of risk factors for CRKP infection.

Variables	CRKP	CSKP	Univariate analysis		Multivariate analysis	
	(n=40)	(n=42)	OR (95% CI)	P value	OR (95% CI)	P value
Age (years)	66.0 (55.0,77.0)	61.0 (55.3,70.3)	1.011 (0.983-1.039)	0.460		
Gender (male), n (%)	32 (80)	31 (73.8)	1.419 (0.504-4.000)	0.508		
CCI > 3, n (%)	6 (40.0)	7 (16.7)	3.333 (1.191-9.327)	0.022*	5.489(1.611-18.696)	0.006*
ICU admission, n (%)	24 (60.0)	12 (28.6)	3.750 (1.493-9.420)	0.005*		
PITT bacteremia score	4.5 (2.0,9.75)	1.0 (0,6.0)	1.139 (1.029-1.261)	0.012*		
Invasive procedures, n (%)						
Mechanical ventilation	26 (65.0)	17 (40.5)	2.731 (1.115-6.687)	0.028*		
Central venous catheter	24 (60.0)	17 (40.5)	2.206 (0.912-5.334)	0.079		
Urinary catheter	29 (72.5)	21 (50.0)	2.636 (1.050-6.620)	0.039*		
Drainage tube	22 (55.0)	24 (57.1)	0.917 (0.383-2.194)	0.845		
Blood transfusion	20 (50.0)	10 (23.8)	3.20 (1.247-8.213)	0.016*		
surgery	17 (42.5)	16 (38.1)	1.201 (0.496-2.906)	0.684		
Infection type, n (%)						
Respiratory tract infections	28 (70.0)	11 (26.2)	6.576 (2.506-17.253)	<0.001*	9.096(3.055-27.082)	<0.001*
Urinary tract infections	3 (7.5)	5 (11.9)	0.600 (0.134-2.695)	0.505		
Hepatobiliary infection	3 (7.5)	10 (23.8)	0.259 (0.066-1.025)	0.054		
Prior use of carbapenems before *K. pneumoniae* isolation, n (%)						
Cephalosporins	8 (20.0)	13 (31.0)	0.558 (0.202-1.537)	0.259		
β-lactam/β-lactamase inhibitors	14 (35.0)	12 (28.6)	1.346 (0.530-3.422)	0.532		
Carbapenems	25 (62.5)	17 (40.5)	2.451 (1.008-5.959)	0.048*		
Hospital-acquired infection, n (%)	34 (85.0)	24 (57.1)	4.250 (1.470-12.285)	0.008*		

*P<0.05; CCI, Charlson comorbidity index; ICU, Intensive Care Unit; P values in the univariate analysis were obtained from univariate logistic regression models. Variables with P < 0.05 were entered into the multivariable logistic regression model using the forward likelihood ratio (Forward LR) method. Odds ratios (ORs), 95% confidence intervals (CIs), and P values were calculated.

### 3.2 Antibiotic resistance and resistance genes

Significant differences in the antibiotic resistance profiles and resistance gene carriage were observed between the CRKP and CSKP groups (**Table 2**). Isolate-level antimicrobial susceptibility results and AMR gene profiles are provided in **Supplementary Tables 5**, **S6**. Overall, a general concordance between resistance phenotypes and corresponding resistance genes was observed across most antibiotic classes, with CRKP isolates showing higher resistance rates and higher prevalence of associated resistance determinants. However, a discrepancy was noted in quinolone resistance (**Table 2**). Although phenotypic resistance to ciprofloxacin and levofloxacin was higher in CRKP isolates, plasmid-mediated quinolone resistance (PMQR) genes (*qnrS1/S5、qnrB2/B31*、aac(6’)-Ib-cr、*oqxA/B*) were highly prevalent in both groups, particularly in CSKP isolates. In contrast, chromosomal quinolone resistance-associated genes (*gyrA* and *parC*) were significantly more prevalent in CRKP isolates than in CSKP isolates. In addition, most CRKP strains (n = 37) harbored the carbapenemase gene *blaKPC-2*, but no other carbapenemase genes were detected. In contrast, none of the CSKP strains harbored any carbapenemase genes. The porin-associated gene *ompK37* was detected in all CRKP and CSKP isolates (**Table 2**).

**Table 2:** Antimicrobial resistance phenotypes and resistance gene profiles of CRKP and CSKP isolates.

Antibiotic classes	Resistance genes	Resistance phenotypes, n (%)			Resistance genes, n (%)		
		CRKP	CSKP	P value	CRKP	CSKP	P value
Carbapenems	*blaKPC-2*	40 (100.0)	0	<0.001*	37 (92.5)	0	<0.001*
	*ompK37*				40 (100.0)	42 (100.0)	1.000
β-lactams	*blaSHV* (mainly *blaSHV-187*)	40 (100.0)	12 (28.6)	<0.001*	40 (100.0)	38 (90.5)	0.137
	*blaTEM* (mainly *blaTEM-1*)				37 (92.5)	6 (14.3)	<0.001*
	*blaCTX-M* (mainly *blaCTX-M-65*)				36 (90.0)	8 (19.0)	<0.001*
	*blaOXA*				6 (15.0)	3 (7.1)	0.433
Quinolones	*qnrS1/S5, qnrB2/B31, aac(6’)-Ib-cr, oqxA/B*	40 (100.0)	8 (19.0)	<0.001*	38 (95.0)	41 (97.6)	0.966
	*gyrA, parC*				40 (100.0)	2(4.8)	<0.001*
Aminoglycosides	*rmtB, armA, aadA2/A3/A16/A24, aac(6’)-Ib*, etc.	36 (90.0)	1 (2.4)	<0.001*	37 (92.5)	8 (19.0)	<0.001*
Tetracyclines	*tetA/B/D/R*	29 (72.5)	9 (21.4)	<0.001*	30 (75.0)	10 (23.8)	<0.001*
Sulphonamides	*sul*	36 (90.0)	4 (9.5)	<0.001*	40 (100.0)	14 (33.3)	<0.001*
Trimethoprim	*dfrA*	36 (90.0)	4 (9.5)	<0.001*	37 (92.5)	7 (16.7)	<0.001*
Chloramphenicol	*floR, cat*	28 (70.0)	10 (23.8)	<0.001*	26 (65.0)	6 (14.3)	<0.001*
Fosfomycin	*fosA*	–	–	–	40 (100.0)	42 (100.0)	1.000
Macrolides	*mphA/E/I*	–	–	–	6 (15.0)	7 (16.7)	0.836
Rifampin	*arr-3*	–	–	–	3 (7.5)	2 (4.8)	0.955
Ceftazidime/Avibactam	–	6 (15.0)	0	0.029	–	–	–
Colistin	–	2 (5.0)	0	0.235	–	–	–

*P<0.05; Resistance phenotype percentages were calculated as the number of isolates resistant to each antimicrobial agent divided by the total number of isolates in the corresponding group. Resistance gene percentages were calculated as the number of isolates carrying a specific resistance gene divided by the total number of isolates in the corresponding group. Differences between CRKP and CSKP groups were analysed using Pearson’s chi-square test or Fisher’s exact test, as appropriate.

### 3.3 Virulence genes and plasmid replicon profiling

All *K. pneumoniae* strains carried virulence genes encoding the siderophore enterobactin (*entA/B* and *fepC/G*). CRKP strains harbored significantly more virulence genes encoding siderophores, such as yersiniabactin (*ybtA/E/P/Q/S/T/U/X*, *irp1/2*, *fyuA*; 97.5% vs. 52.4%, *P* < 0.001) and aerobactin (*iutA* and *iucA/B/C*; 92.5% vs. 38.1%, *P* < 0.001) but fewer genes encoding salmochelin (*iroB/C/D/N*; 5.0% vs. 45.2%, *P* < 0.001) than CSKP isolates. Moreover, virulence genes related to pili (*ykgK/ecpR* and *yagZ/ecpA*), outer membrane proteins (*ompA*), and heat-stable enterotoxins (*astA*) were detected in the *K. pneumoniae* strains (**Table 3**).

**Table 3:** Virulence genes in CRKP and CSKP groups.

Virulence factors		Virulence genes	CRKP, n (%)	CSKP, n (%)	*P* value
siderophores	enterobactin	*entA/B, fepC/G*	40 (100.0)	42 (100.0)	1.000
	yersiniabactin	*ybtA/E/P/Q/S/T/U/X, irp1/2, fyuA*	39 (97.5)	22 (52.4)	<0.001*
	aerobactin	*iutA, iucA/B/C*	37 (92.5)	16 (38.1)	<0.001*
	salmochelin	*iroB/C/D/N*	2 (5.0)	19 (45.2)	<0.001*
pili		*ykgK/ecpR, yagZ/ecpA*, etc.	39 (97.5)	42 (100.0)	0.488
outer membrane proteins		*ompA*	40 (100.0)	42 (100.0)	1.000
heat-stable enterotoxins		*astA*	2 (5.0)	1 (2.4)	0.966

*P<0.05; Virulence gene percentages were calculated as the number of isolates carrying each virulence-associated gene divided by the total number of isolates in the corresponding group. Differences in virulence gene distribution between CRKP and CSKP groups were evaluated using Pearson’s chi-square test or Fisher’s exact test, as appropriate.

**Figure 1:**
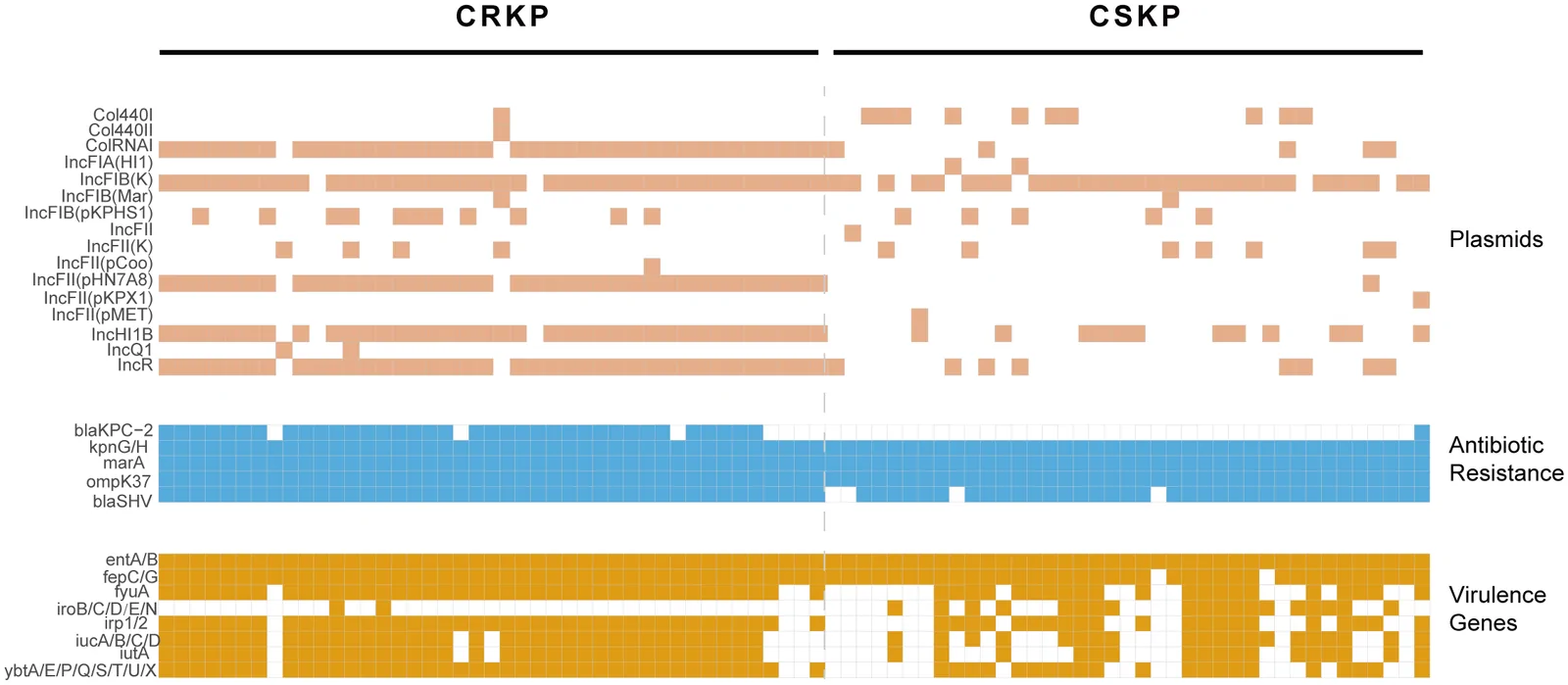
Distribution of plasmid replicons, antibiotic resistance genes, and virulence genes in CRKP and CSKP isolates. Heatmap showing the presence (colored squares) and absence (white squares) of detected plasmid replicons, antimicrobial resistance genes, and virulence genes across individual isolates. The heatmap illustrates differences in replicon and gene carriage patterns between CRKP and CSKP groups. Plasmid replicon profiles represent detected replicon types and do not indicate complete plasmid structures or direct linkage between replicons and specific resistance or virulence determinants.

Comparative analysis of plasmid replicon profiles revealed a marked difference between CRKP and CSKP (**Figure 1**). CRKP isolates showed a higher prevalence of multi-replicon profiles, with IncFII/IncFIB, IncR, and IncHI1B replicons frequently detected, whereas CSKP isolates were mainly characterized by Col-type replicons. Several IncFII/IncFIB-positive isolates also harbored *blaKPC-2*, reflecting their frequent co-occurrence in CRKP isolates. However, replicon-based analysis alone cannot determine the complete plasmid structure or establish direct genetic linkage between specific replicons and resistance or virulence determinants.

### 3.4 Phylogenetic analysis

**Figure 2:**
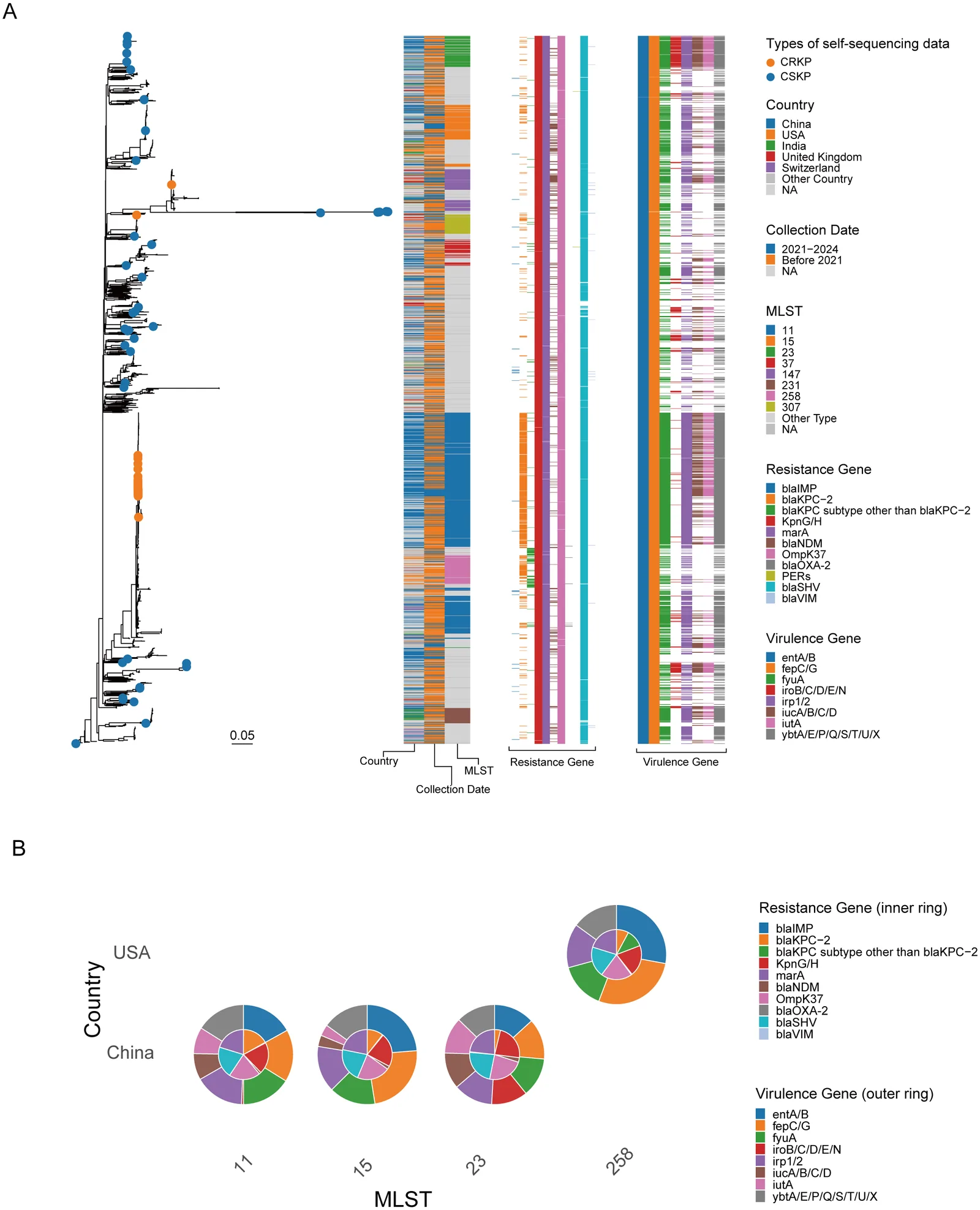
Phylogenetic relationships and genomic features of global *Klebsiella pneumoniae* isolates. **(A)** Phylogenetic tree illustrating the evolutionary relationships among *K. pneumoniae* isolates. Metadata displayed alongside the tree include country of origin, collection date, multilocus sequence type (MLST), resistance genes, and virulence genes. Presence and absence of genes are indicated by colored and white squares, respectively. **(B)**Dual-layer pie charts showing the geographic distribution and genetic profiles of selected major sequence types (ST11, ST15, ST23, and ST258; n > 40). Each pie chart represents one sequence type, with country origin indicated by the corresponding geographic labels. The inner ring represents resistance-associated genes, and the outer ring represents virulence-associated genes. Additional sequence types and genetic profiles are provided in **Supplementary Tables 7** and **S8**.

A phylogenetic tree was constructed based on 2,460 publicly available *K. pneumoniae* genomes and 82 isolates from this study (40 CRKP and 42 CSKP) (**Figure 2A**). These publicly available genomes were obtained from 60 countries or regions across five continents, with China, the United States, India, the United Kingdom, and Italy contributing the largest numbers of genomes. Multiple sequence types (STs) were identified, including ST11, ST15, ST147, ST258, ST23, ST307, ST37, and ST231. Among the 40 CRKP isolates obtained from our hospital, 38 belonged to ST11, while the rest belonged to ST307 and ST147. As shown in **Figure 2**, most CRKP isolates formed a distinct phylogenetic cluster, whereas CSKP isolates were distributed across multiple branches, indicating greater phylogenetic diversity.

Within the publicly available genome dataset, distinct geographic distributions of STs and carbapenemase genotypes were observed. Among the major STs visualized in **Figure 2B**, ST11, ST15, ST23, and ST258 showed different geographic distributions, with ST11, ST15, and ST23 frequently identified among genomes from China and ST258 predominantly observed among genomes from the United States. Analysis of the complete dataset further revealed that ST231 was mainly identified among genomes from India (**Supplementary Table 7**). Distinct STs showed different associations with carbapenemase genotypes. ST11, ST258, ST23, and ST307 were frequently associated with *blaKPC-2*, whereas ST15, ST147, and ST37 showed higher frequencies of *blaNDM* carriage (**Supplementary Table 8**). The number of *K. pneumoniae* strains harboring genes encoding enterobactin was the highest, followed by those with genes encoding yersiniabactin and aerobactin, while the carriage rate of genes encoding salmochelin was the lowest (**Supplementary Table 9**). ST23 strains possessed all four siderophore-encoding gene clusters (**Supplementary Table 10**).

### 3.5 Pan-genome analysis

**Figure 3:**
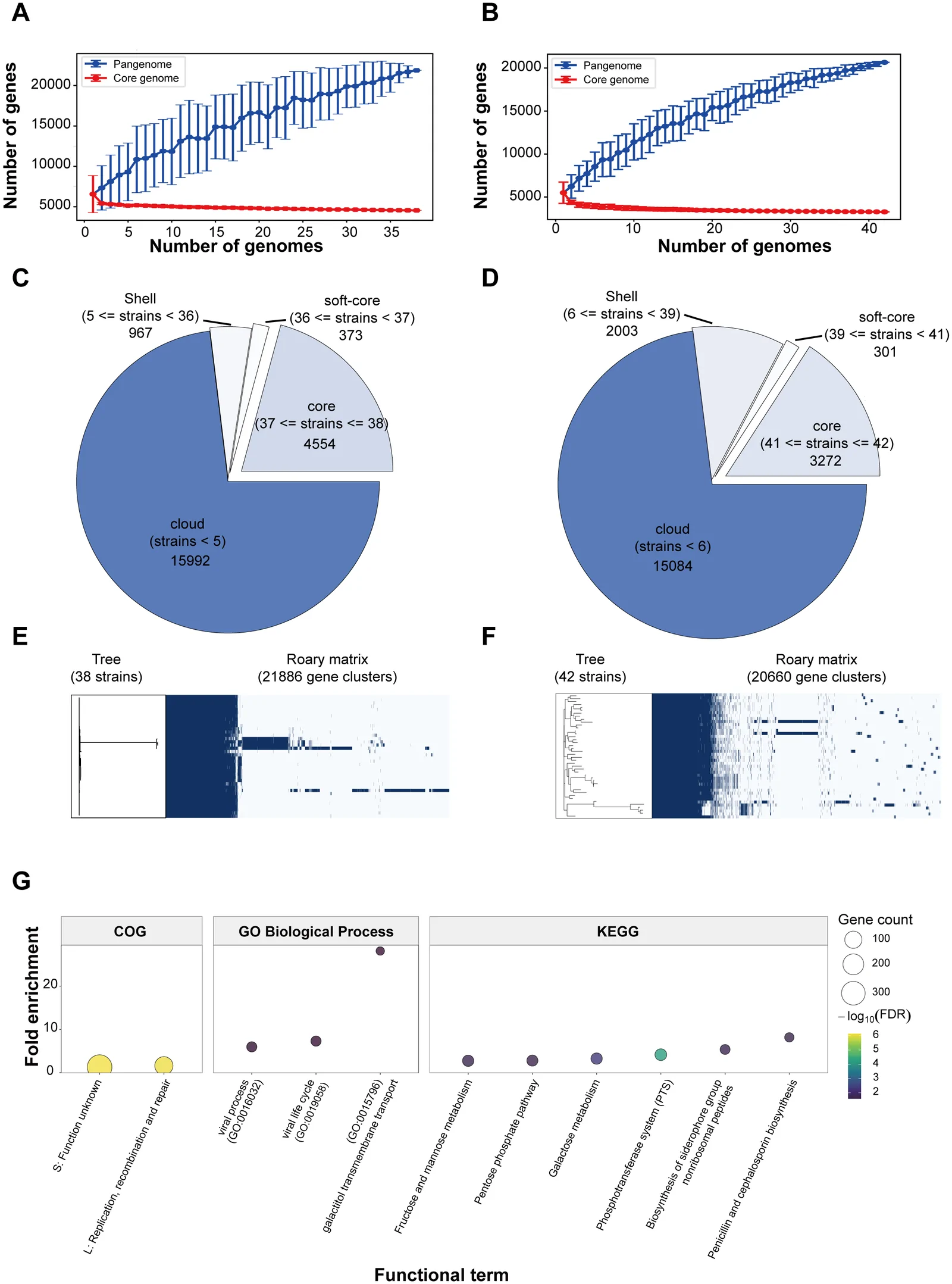
Pangenome analysis of ST11 CRKP and CSKP isolates. **(A, B)** Pangenome and core genome accumulation curves with error bars for ST11 CRKP **(A)** and CSKP **(B)** strains, showing gene discovery trends as additional genomes are sequentially added. The plateau of the core genome curve indicates genomic conservation, whereas the continuously increasing pangenome curve reflects genomic diversity. **(C, D)** Distribution of core, soft-core, shell, and cloud genes in ST11 CRKP **(C)** and CSKP **(D)**, illustrating the proportion of conserved versus accessory genes within each population. **(E, F)** Whole-genome phylogenetic trees integrated with gene presence/absence matrices, where blue indicates gene presence and white indicates absence, demonstrating the relationship between phylogeny and accessory genome composition in ST11 CRKP **(E)** and CSKP **(F)**. **(G)** ctional enrichment analysis of CRKP-associated accessory genes across COG categories, GO Biological Process terms, and KEGG pathways. Bubble size represents gene count, bubble colour indicates -log10(FDR), and the y-axis shows fold enrichment.

Pan-genome analysis of the ST11 CRKP and CSKP groups revealed extensive genetic diversity (**Figure 3**). As the number of genomes increased, the pan-genome size increased, while the number of core genes decreased and eventually stabilized, reflecting open pan-genome characteristics in both groups (**Figures 3A, B**). Genes were categorized into core (≥99%), soft-core (95–99%), shell (15–95%), and cloud (<15%) groups (**Figures 3C, D**), with cloud genes constituting the largest portion of the pan-genome in both groups. Whole-genome phylogenetic trees integrated with gene presence/absence matrices (**Figures 3E, F**) further highlighted the genomic landscape, revealing that the ST11 CRKP group contained slightly more core genes than the CSKP group.

Differential accessory-gene analysis identified a set of genes strongly associated with CRKP. For example, *ybiI_3*, annotated as putative protein YbiI, was present in 39 of 40 CRKP isolates but absent from all 42 CSKP isolates. Several additional accessory gene clusters, including *xerC_1*, annotated as tyrosine recombinase XerC, and *intS_1*, annotated as prophage integrase IntS, were present in 38 of 40 CRKP isolates and absent from all CSKP isolates (**Supplementary Table 11**). These genes showed large prevalence differences between CRKP and CSKP and remained significant after FDR correction. Functional annotation showed that many CRKP-associated accessory genes encoded hypothetical proteins, whereas others were related to recombination or mobile genetic elements, including tyrosine recombinases and prophage integrases. Together, these findings suggest that ST11 CRKP isolates carried a set of highly prevalent accessory genomic components that were largely absent from CSKP isolates, with signatures of mobile-element-associated and recombination-associated regions.

Consistent with these gene-level observations, functional enrichment analysis showed that CRKP-associated accessory genes clustered in several broader functional categories and pathways (**Figure 3G**). COG analysis showed significant enrichment of categories related to function unknown and replication, recombination and repair, while GO Biological Process analysis highlighted viral process, viral life cycle and galactitol transmembrane transport. KEGG analysis further identified enrichment of pathways involved in carbohydrate metabolism and transport, including fructose and mannose metabolism, the pentose phosphate pathway, galactose metabolism and the phosphotransferase system, together with siderophore-group nonribosomal peptide biosynthesis and penicillin and cephalosporin biosynthesis. These results extend the gene-level findings and suggest that CRKP-associated accessory genome differences involve broader functional patterns related to genome plasticity, metabolic adaptation, nutrient acquisition and antimicrobial stress responses.

### 3.6 SNV and InDel analysis

**Figure 4:**
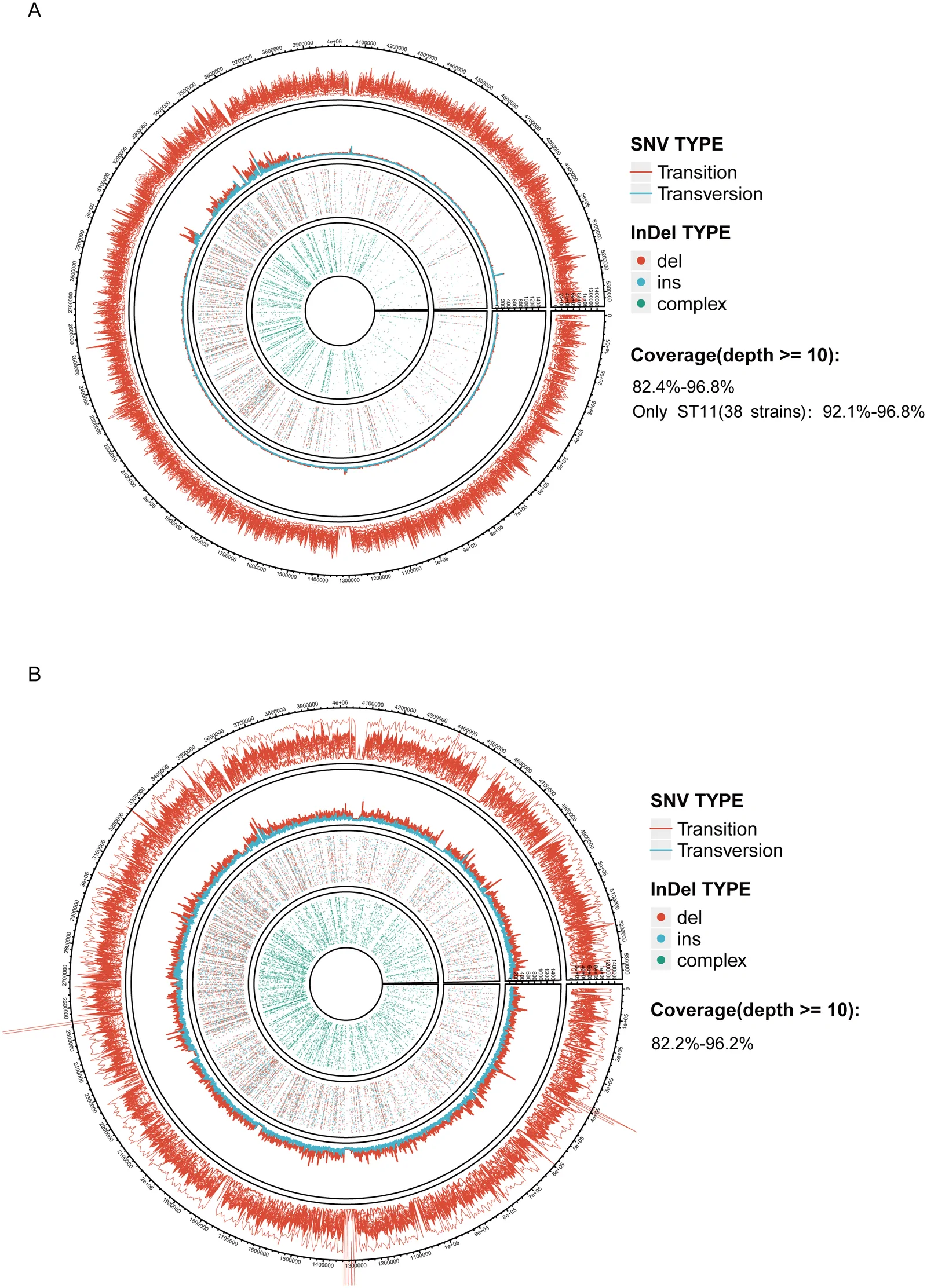
SNV and InDel analysis of CRKP and CSKP isolates. **(A, B)** Circular diagrams illustrating the distribution of genetic variations in CRKP **(A)** and CSKP **(B)** strains. From outer to inner rings, tracks represent genome coverage, sequencing depth, SNV distribution, and InDel events, respectively, allowing visualization of genomic variation patterns across isolates.

**Figure 5:**
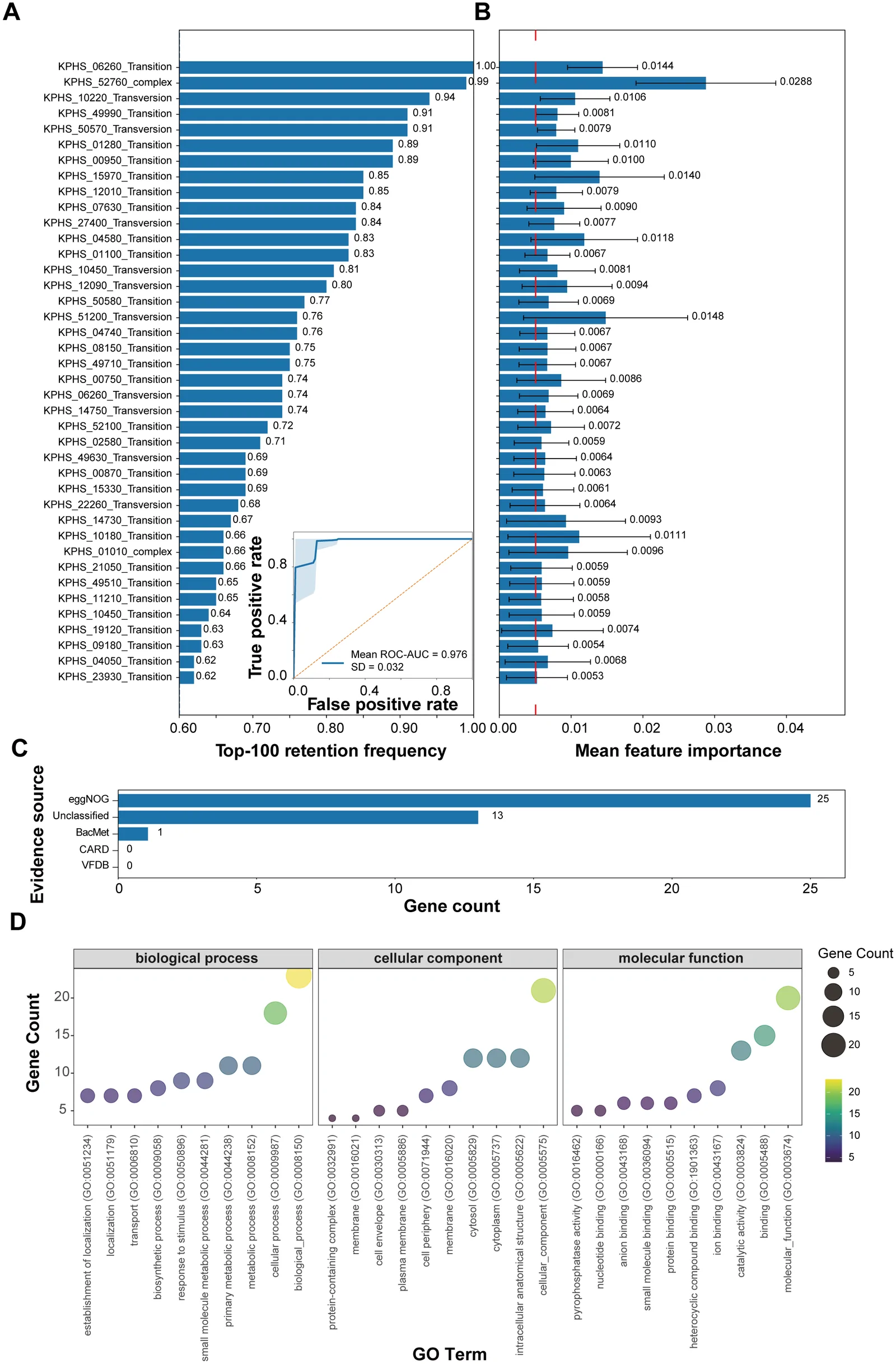
Functional annotation of high-importance SNV/InDel-associated loci distinguishing CRKP from CSKP isolates. **(A)** Stability of prioritized SNV/InDel-associated features across 100 repeated cross-validation evaluations, shown as top-100 retention frequency. The inset shows the mean ROC curve across cross-validation evaluations, with a mean ROC-AUC of 0.976 and SD of 0.032. **(B)** Mean Random Forest feature importance of the stable features across repeated cross-validation evaluations; error bars indicate variability in feature importance, and the red dashed line marks a mean importance of 0.005. **(C)** Annotation evidence sources for the prioritized SNV/InDel-associated loci, including eggNOG, BacMet, CARD, and VFDB, together with unclassified loci. **(D)** Gene Ontology annotation of eggNOG-annotated loci across the biological process, cellular component, and molecular function domains. Bubble size and colour represent the number of genes assigned to each GO term.

Genome-wide comparison of SNVs and InDels between CRKP and CSKP isolates revealed distinct distribution patterns (**Figure 4**). In CRKP isolates, SNV and InDel signals were relatively concentrated in specific genomic regions, particularly around approximately 3.05-3.10 Mb and 3.23-3.74 Mb, whereas CSKP isolates showed a more dispersed distribution across the genome. To further prioritize genomic regions potentially associated with CRKP-related variation, we applied a Random Forest classifier to distinguish CRKP from CSKP isolates. The model showed robust and reproducible discriminatory performance. Across repeated stratified five-fold cross-validation, it achieved a mean ROC-AUC of 0.976 with a standard deviation of 0.032, indicating consistently high predictive performance across repeated data partitions (**Figure 5A**). Feature-stability analysis across the 100 cross-validation evaluations identified 40 features with top-100 retention frequencies greater than 0.60 (**Figure 5A**). This threshold was selected to balance feature stability across repeated data partitions with the retention of a sufficient number of candidate proteins for downstream functional annotation and pathway-level analysis. Notably, all 40 stable features also had mean importance values greater than 0.005 (**Figure 5B**).

Integrated annotation of the prioritized SNV/InDel-associated loci identified no CARD-defined resistance genes or VFDB-defined virulence genes, whereas one locus was associated with metal or biocide tolerance based on BacMet annotation. Twenty-five loci were functionally annotated using eggNOG, while 13 remained unclassified (**Figure 5C**; **Supplementary Table 12**). As a complementary descriptive classification, the annotated loci were also grouped according to putative adaptive or fitness-related functions, with the most frequent categories involving cell envelope or membrane functions, transport, efflux or secretion, stress response or protein quality control, and DNA repair, recombination or replication (**Supplementary Table 12**). Further GO annotation provided a more systematic functional overview across the three major GO domains. Biological process annotations were mainly associated with cellular and metabolic processes, transport and localization, responses to stimuli, and biosynthetic processes; cellular component annotations were predominantly related to cytoplasmic, membrane, cell-envelope, and cell-periphery components; and molecular function annotations were largely associated with binding and catalytic activities (**Figure 5D**).

Together, these results indicate that the SNV/InDel features distinguishing CRKP from CSKP were associated with a broad range of cellular and molecular functions rather than canonical CARD-defined resistance genes. These loci should therefore be regarded as candidate genomic features associated with CRKP–CSKP differentiation and warrant further functional validation.

## 4 Discussion

This study provides a comprehensive analysis of the clinical and genomic landscape of CRKP in a tertiary hospital setting. We identified a CCI > 3 and respiratory tract infection as independent risk factors for CRKP infection. Genomic analyses demonstrated that CRKP isolates carried significantly more resistance and virulence determinants than CSKP, with ST11-*blaKPC-2* representing the predominant epidemic clone. Phylogenetic analysis further revealed distinct geographic distributions of major sequence types and carbapenemase genotypes, highlighting regional differences in CRKP dissemination. In addition, pan-genome analysis revealed substantial genomic plasticity, while comparative SNV/InDel analyses identified genomic features associated with diverse cellular, metabolic, transport, and membrane-related functions rather than canonical resistance genes. Together, these findings provide a comprehensive understanding of the clinical characteristics, population structure, and evolutionary mechanisms underlying the emergence and dissemination of CRKP.

A CCI > 3 and respiratory tract infections were identified as independent risk factors for CRKP infection. A CCI > 3 reflects a greater burden of chronic comorbidities, which often compromises immune function and increases susceptibility to opportunistic infections (Liu et al., 2021). Respiratory tract infections may serve as a risk factor because of the need for invasive procedures, such as sputum suction, bronchoscopy, intubation, and mechanical ventilation, all of which disrupt mucosal barriers and facilitate pathogen transmission (Cohen et al., 2024). Therefore, for patients with a CCI > 3 and respiratory tract infections, enhanced bacterial surveillance and optimized antimicrobial stewardship are warranted.

In this study, the CRKP isolates exhibited higher resistance rates and carried a broader array of resistance genes than CSKP isolates. The *blaKPC-2* gene was the most frequently detected carbapenemase gene, indicating that *blaKPC-2*-mediated carbapenemase production is likely the predominant carbapenem resistance mechanism in our hospital (Liao et al., 2020; Wang et al., 2024). Notably, three CRKP isolates lacked detectable carbapenemase genes, indicating that additional resistance mechanisms may be involved. Although *ompK37* was identified in all isolates, its ubiquitous presence suggests that it does not account for the observed carbapenem resistance phenotype. As alterations in the major porins *ompK35* and *ompK36*, as well as porin expression levels, were not assessed, the role of non-carbapenemase-mediated resistance could not be fully determined. This represents a limitation of the current study and warrants further investigation. Additionally, the genotype–phenotype relationship for quinolone resistance proved complex. Although PMQR genes were highly prevalent in CSKP, this did not result in high resistance rates to ciprofloxacin and levofloxacin. These genes are known to confer only low-level resistance and may not be sufficient to produce clinically significant resistance (Hooper and Jacoby, 2015). In contrast, gyrA and parC were more frequently detected in CRKP isolates, suggesting their potential contribution to the higher quinolone resistance phenotype. However, specific quinolone resistance-determining region (QRDR) mutations and their functional effects were not evaluated in this study.

Enterobactin was ubiquitous in *K. pneumoniae* strains. ST23 strains harbored more siderophore-associated virulence genes, aligning with their designation as hypervirulent clones (Zhang et al., 2019). Although CRKP isolates possessed more virulence-associated genes than CSKP isolates, this genomic-level difference is not necessarily predictive of virulence phenotype and warrants further experimental validation. The enrichment of IncFII/IncFIB, IncR, and IncHI1B replicons in CRKP is consistent with previous reports in which IncFII/IncFIB plasmids have frequently been associated with *blaKPC* carriage and efficient plasmid maintenance and conjugation systems (Jiang et al., 2025). In contrast, the predominance of small Col-type replicons in CSKP is consistent with relatively lower plasmid complexity. Overall, these findings suggest that differences in replicon composition may be associated with the genomic characteristics of CRKP. However, plasmid reconstruction was not performed; therefore, direct linkage between specific replicon types and resistance or virulence genes cannot be established based on replicon analysis alone.

Herein, ST11 was the dominant clone among the CRKP isolates and predominantly clustered with strains from China, consistent with the reported predominance of ST11 in China (Wang et al., 2022; Shi et al., 2024; Wang et al., 2024). Additionally, one ST307 isolate and one ST147 isolate clustered with publicly available genomes from outside China, suggesting possible epidemiological links that warrant further investigation. Compared to CSKP, CRKP showed marked clonal clustering, consistent with previous reports (Zhang et al., 2022). Comparative analysis of publicly available genomes identified regional differences in ST distribution and carbapenemase genotypes, consistent with previous reports (Wang et al., 2024). However, these geographic patterns should be interpreted cautiously. The public genomic dataset was restricted to high-quality complete or chromosome-level RefSeq assemblies to improve analytical consistency, but this strategy may reduce the representation of global *K. pneumoniae* diversity. Furthermore, publicly available genome collections are inherently influenced by uneven geographic representation, differences in sequencing capacity and surveillance priorities, and preferential deposition of clinically important or outbreak-associated isolates. Therefore, the observed phylogenetic patterns should be considered a comparative genomic framework for contextualizing the study isolates rather than a comprehensive representation of the global population structure of *K. pneumoniae*. These findings provide insights into regional clonal diversity and highlight the importance of continued surveillance of CRKP lineages and resistance determinants.

Pan-genome analysis revealed that the pan-genome of *K. pneumoniae* isolates was open, and cloud genes comprised the largest portion of the genome, indicating high strain-specific gene diversity, in agreement with the findings of Munim et al. (2024). Notably, ST11 CRKP isolates contained slightly more core genes than CSKP isolates, indicating differences in core genome composition between the two groups (Wyres et al., 2020). Differential accessory-gene analysis identified several CRKP-associated genes, including *ybiI*, *xerC*, and *intS*, which were highly prevalent in CRKP but absent from CSKP isolates. Functional annotation showed that these genes were mainly associated with mobile genetic elements and recombination-related functions (Midonet and Barre, 2014; Shen et al., 2020). Furthermore, pathway-level enrichment analyses revealed that CRKP-associated accessory genome differences involve multiple biological functions, including genome plasticity, metabolic processes, nutrient acquisition, and stress-related functions. However, the specific contributions of these accessory genes and enriched pathways to CRKP phenotypes require further experimental validation.

Genome-wide comparison revealed distinct SNV and InDel distribution patterns between CRKP and CSKP isolates, with discriminatory variants concentrated in specific genomic regions. These patterns may be consistent with differences in genomic background and bacterial physiology, although the present comparative analysis cannot establish adaptive evolution or antimicrobial selection as their direct cause. To complement single-locus comparative analyses, the Random Forest framework provided a multivariate approach for prioritizing SNV/InDel-associated features according to their joint contribution to CRKP-CSKP discrimination. By evaluating multiple genomic features simultaneously, the model can capture discriminatory signals embedded within correlated and potentially nonlinear feature patterns, while repeated cross-validation and feature-stability analysis enabled prioritization of loci that remained consistently informative across different data partitions. Notably, the prioritized loci included no CARD-defined resistance genes or VFDB-defined virulence genes, whereas one locus was associated with metal or biocide tolerance. Broader eggNOG and GO annotation showed that the prioritized loci were distributed across diverse cellular and molecular functions, including cellular and metabolic processes, transport and responses to stimuli, membrane- and cell-envelope-associated components, and binding and catalytic activities. These observations are consistent with the possibility that CRKP-CSKP differentiation involves broader physiological and genomic differences beyond canonical acquired resistance determinants (Dong et al., 2025; Mmatli et al., 2025), but they do not demonstrate that the identified loci directly mediate resistance or adaptive evolution. Moreover, Random Forest feature importance reflects predictive contribution rather than biological causality, and feature rankings may be influenced by correlations among variants, genetic linkage, and population structure. The prioritized loci should therefore be regarded as reproducible, hypothesis-generating candidates that warrant further functional and experimental validation.

This study has several limitations. First, its retrospective single-center design and relatively small sample size may limit the generalizability of the findings and affect the stability of the multivariable regression and Random Forest analyses. Although internal validation procedures, including model calibration, discrimination assessment, repeated cross-validation, and feature-stability analysis, were used to improve robustness, the identified predictors and genomic features require further validation in larger independent cohorts. Second, our conclusions were primarily based on comparative genomic analyses without functional validation. The candidate loci identified in this study, as well as the mechanisms underlying carbapenem resistance in non-carbapenemase-producing CRKP isolates, require further investigation. In addition, database-dependent annotations, including virulence factor identification, may vary with updates to reference databases. Therefore, the reported virulence profiles should be interpreted within the context of the database versions used in this study. Furthermore, plasmid reconstruction was not performed, preventing detailed characterization of the genetic contexts, transmission dynamics, and co-localisation patterns of resistance and virulence determinants. Future multicentre studies incorporating larger cohorts, long-read sequencing, plasmid reconstruction, transcriptomic analyses, and functional experiments will be valuable for validating these findings and further elucidating CRKP evolution, resistance mechanisms, and dissemination.

## 5 Conclusion

In conclusion, this study integrated clinical and genomic analyses to characterize CRKP isolates from a tertiary hospital in Wuhan, China. A Charlson Comorbidity Index >3 and respiratory tract infections were identified as independent risk factors for CRKP infection. ST11 carrying *blaKPC-2* represented the predominant CRKP lineage, and CRKP isolates exhibited higher carriage of antimicrobial resistance determinants and siderophore-associated genes compared with CSKP isolates. Comparative genomic analyses revealed differences in plasmid replicon profiles, accessory genome composition, and SNV/InDel distribution between CRKP and CSKP isolates, providing insights into the genomic characteristics associated with CRKP. Although further functional validation is required, these findings improve the understanding of the clinical characteristics, clonal distribution, and genomic diversity of CRKP and may support future surveillance and infection-control strategies.

## Data Availability

The datasets presented in this study can be found in online repositories. The names of the repository/repositories and accession number(s) can be found below: https://ngdc.cncb.ac.cn/bioproject/browse/PRJCA040206, PRJCA040206.
