# Supplementary material for: Integrative genomic and clinical analysis of carbapenem-resistant and susceptible Klebsiella pneumoniae isolates from a tertiary hospital in Wuhan
Source: Front Cell Infect Microbiol. 2026 Aug 12;16:1881548. doi: 10.3389/fcimb.2026.1881548 (PMC13506390; doi:10.3389/fcimb.2026.1881548)
Supplement: Supplementary file 1 [file SupplementaryFile1.docx]

***Supplementary material***

Supplementary Tables S1-S3, S5, S6, S11, and S12 are provided as separate Excel files to facilitate data accessibility and reproducibility. The files were automatically renamed by the submission system according to upload order; therefore, the table numbers in the file names may differ from the labels used in the manuscript.

**Table S1**(provided as a separate Excel file)**.** Raw-read quality assessment metrics generated by FastQC before quality filtering for all *K. pneumoniae* isolates. The table includes FastQC module status, GC content, sequence length and total sequence counts for each isolate.

**Table S2**(provided as a separate Excel file)**.** De novo genome assembly quality metrics after fastp-based quality filtering for all *Klebsiella pneumoniae* isolates. Assembly statistics include total assembly length, GC content, number of contigs, N50 and related contiguity metrics generated using SPAdes v3.15.5.

**Table S3**(provided as a separate Excel file)**.** Raw clinical and demographic data of patients with *K. pneumoniae* infection used for statistical analyses.

**Table S4.** Clinical and demographic characteristics of patients with K. pneumoniae infection and statistical comparisons between CRKP and CSKP groups.

**Table S5**(provided as a separate Excel file)**.** Isolate-level antimicrobial susceptibility testing results for all antibiotics, reported as susceptible (S), intermediate (I), or resistant (R) according to CLSI M100 (30th edition, 2020).

**Table S6**(provided as a separate Excel file)**.** Isolate-level antimicrobial resistance gene profiles identified by whole-genome sequencing.

**Table S7.** Geographic distribution of major sequence types among global *K. pneumoniae* isolates included in the comparative phylogenetic analysis.

**Table S8.** Distribution of carbapenemase genes among major sequence types of global *K. pneumoniae* isolates.

**Table S9.** Distribution of siderophore-associated virulence genes among global *K. pneumoniae* isolates.

**Table S10.** Distribution of siderophore-associated virulence gene clusters across major sequence types of global *K. pneumoniae* isolates.

**Table S11**(provided as a separate Excel file)**.** Accessory gene association analysis between CRKP and CSKP isolates, including all tested accessory genes, significantly CRKP-enriched genes, and functional enrichment results.

**Table S12**(provided as a separate Excel file)**.** Integrated annotation of CRKP-associated SNV/InDel loci using CARD, VFDB, BacMet2, and eggnog, including integrated loci, database summary, adaptive summary, and adaptive keywords.

**Table S4** Clinical and demographic characteristics of patients with K. pneumoniae infection and statistical comparisons between CRKP and CSKP groups.

| Variables | Total (n = 82) | CRKP (n = 40) | CSKP (n = 42) | *P* value |  |
| --- | --- | --- | --- | --- | --- |
| Age (years) | 62.0 (55.0, 74.0) | 66.0 (55.0, 77.0) | 61.0 (55.25, 70.25) | 0.444 |  |
| Gender (male), n (%) | 63 (76.8) | 32 (80) | 31 (73.8) | 0.507 |  |
| Comorbidities, n (%) | | | | | |
| Cardiovascular disease | 40 (48.8) | 25 (62.5) | 15 (35.7) | 0.015* |  |
| Chronic lung disease | 12 (14.6) | 8 (20.0) | 4 (9.5) | 0.180 |  |
| Chronic liver disease | 9 (11.0) | 6 (15.0) | 3 (7.1) | 0.433 |  |
| Chronic renal disease | 13 (15.9) | 9 (22.5) | 4 (9.5) | 0.108 |  |
| Solid tumors or hematologic malignancies | 16 (19.5) | 8 (20.0) | 8 (19.0) | 0.913 |  |
| Diabetes | 22 (26.8) | 13 (32.5) | 9 (21.4) | 0.258 |  |
| Neurological disorders | 29 (35.4) | 16 (40.0) | 13 (31.0) | 0.392 |  |
| CCI > 3, n (%) | 23 (28.0) | 16 (40.0) | 7 (16.7) | 0.019* |  |
| ICU admission, n (%) | 36 (43.9) | 24 (60.0) | 12 (28.6) | 0.004* |  |
| PITT bacteremia score | 2.0 (0-8.0) | 4.5 (2.0, 9.75) | 1.0 (0, 6.0) | 0.03* |  |
| Invasive procedures, n (%) | | | | | |
| Mechanical ventilation | 43 (52.4) | 26 (65.0) | 17 (40.5) | 0.026* |  |
| Central venous catheter | 41 (50.0) | 24 (60.0) | 17 (40.5) | 0.077 |  |
| Urinary catheter | 50 (61.0) | 29 (72.5) | 21 (50.0) | 0.037* |  |
| Drainage tube | 46 (56.1) | 22 (55.0) | 24 (57.1) | 0.845 |  |
| Blood transfusion | 30 (36.6) | 20 (50.0) | 10 (23.8) | 0.014* |  |
| surgery | 33 (40.2) | 17 (42.5) | 16 (38.1) | 0.684 |  |
| Infection type, n (%) | | | | | |
| Respiratory tract infections | 39 (47.6) | 28 (70.0) | 11 (26.2) | ＜0.001* |  |
| Urinary tract infections | 8 (9.8) | 3 (7.5) | 5 (11.9) | 0.764 |  |
| Hepatobiliary infection | 13 (15.9) | 3 (7.5) | 10 (23.8) | 0.043* |  |
| Prior use of carbapenems before *K.pneumoniae* isolation, n (%) | | | | | |
| Cephalosporins | 21 (25.6) | 8 (20.0) | 13 (31.0) | 0.256 |  |
| β-lactam/β-lactamase inhibitors | 26 (31.7) | 14 (35.0) | 12 (28.6) | 0.532 |  |
| Carbapenems | 42 (51.2) | 25 (62.5) | 17 (40.5) | 0.046* |  |
| Polymicrobial infection | 27 (32.9) | 12 (30.0) | 15 (35.7) | 0.582 |  |
| Hospital-acquired infection, n (%) | 58 (70.7) | 34 (85.0) | 24 (57.1) | 0.006* |  |
| Clinical Outcomes, n (%) | | | | | |
| Length of stay (days)＞30 | 18 (22.0) | 11 (27.5) | 7 (16.7) | 0.236 |  |
| death | 14 (17.1) | 11 (27.5) | 3 (7.1) | 0.014 |  |

**Notes:** Continuous variables were analysed based on their distribution characteristics. Normally distributed data were expressed as mean ± standard deviation, while non-normally distributed data were expressed as median (interquartile range, IQR). Categorical variables were denoted as frequencies (percentages). Categorical data were analysed using Pearson’s chi-square, Fisher’s exact, or McNemar’s tests. Continuous variables were compared using a two independent samples t-test or Mann–Whitney U test, as appropriate. *P* < 0.05 was considered statistically significant. * *P*＜0.05；CCI: Charlson comorbidity index; ICU: Intensive Care Unit

**Table S7** Geographic distribution of major sequence types among global *K. pneumoniae* isolates included in the comparative phylogenetic analysis

| ST | Country | | | | | | | Total |
| --- | --- | --- | --- | --- | --- | --- | --- | --- |
|  | China | USA | India | United Kindom | Switzerland | Other country | NA |  |
| ST11 | 388 (62.7) | 2 (0.3) | 2 (0.3) | 0 | 12(1.9) | 25 (4.0) | 190 (30.7) | 619 |
| ST15 | 52 (40.9) | 9 (7.1) | 3 (2.4) | 1 (0.7) | 0 | 11 (8.7) | 51 (40.2) | 127 |
| ST23 | 55 (55.6) | 4 (4.0) | 2 (2.0) | 0 | 0 | 13 (13.1) | 25 (25.3) | 99 |
| ST37 | 17 (30.4) | 8 (14.3) | 0 | 1 (1.8) | 1 (1.8) | 8 (14.3) | 21 (37.5) | 56 |
| ST147 | 7 (6.4) | 8 (7.3) | 7 (6.4) | 16 (14.7) | 2 (1.8) | 42 (38.5) | 27 (24.8) | 109 |
| ST231 | 10 (18.5) | 1 (1.9) | 28 (51.9) | 0 | 1 (1.9) | 1 (1.9) | 13 (24.1) | 54 |
| ST258 | 0 | 41 (38.7) | 0 | 1 (0.9) | 1 (0.9) | 24 (22.6) | 39 (36.8) | 106 |
| ST307 | 6 (8.8) | 13 (19.1) | 0 | 8 (11.8) | 8 (11.8) | 14 (20.6) | 19 (27.9) | 68 |

ST: sequence type

**Table S8** Distribution of carbapenemase genes among major sequence types of global *K. pneumoniae* isolates

| ST | Resistance genes | | | | | |
| --- | --- | --- | --- | --- | --- | --- |
|  | *blaKPC-2* | *blaKPC* subtype other than *blaKPC-2* | *blaNDM* | *blaVIM* | *blaIMP* | |
| ST11 | 464 (75.0) | 17 (2.7) | 47 (7.6) | 2 (0.3) | 2 (0.3) | |
| ST15 | 30 (23.6) | 2 (1.6) | 43 (33.9) | 0 | 0 | |
| ST23 | 11 (11.1) | 1 (1.0) | 8 (8.1) | 2 (2.0) | 0 | |
| ST37 | 2 (3.6) | 6 (10.7) | 10 (17.9) | 1 (1.8) | 2 (3.6) | |
| ST147 | 4 (3.7) | 0 | 47 (43.1) | 5 (4.6) | 0 | |
| ST231 | 0 | 0 | 0 | 0 | 0 | |
| ST258 | 64 (60.4) | 37 (34.9) | 1 (0.9) | 0 | 0 |  |
| ST307 | 15 (22.1) | 3 (4.4) | 3 (4.4) | 0 | 1 (1.5) | |

ST: sequence type

**Table S9** Distribution of siderophore-associated virulence genes among global *K. pneumoniae* isolates

| Virulence genes | Proportion, n (%) |
| --- | --- |
| Enterobactin (*entA/B*, *fepC/G*) | 2541 (100.0) |
| Yersiniabactin (*ybtA/E/P/Q/S/T/U/X, irp1/2, fyuA*) | 1493 (58.7) |
| Aerobactin (*iutA, iucA/B/C*) | 696 (27.4) |
| Salmochelin (*iroB/C/D/N*) | 293 (11.5) |

**Table S10** Distribution of siderophore-associated virulence gene clusters across major sequence types of global *K. pneumoniae* isolates

| ST | Virulence genes | | | |
| --- | --- | --- | --- | --- |
|  | Enterobactin (*entA/B*, *fepC/G*) | Yersiniabactin (*ybtA/E/P/Q/S/T/U/X, irp1/2, fyuA*) | Aerobactin (*iutA, iucA/B/C*) | Salmochelin (*iroB/C/D/N*) |
| ST11 | 619 (100.0) | 586 (94.7) | 266 (43.0) | 30 (4.8) |
| ST15 | 127 (100.0) | 87 (68.5) | 18 (14.2) | 0 |
| ST23 | 99 (100.0) | 95 (96.0) | 87 (87.9) | 85 (85.9) |
| ST37 | 56 (100.0) | 18 (32.1) | 3 (5.4) | 0 |
| ST147 | 109 (100.0) | 75 (68.8) | 27 (24.8) | 0 |
| ST231 | 54 (100.0) | 50 (92.6) | 22 (40.7) | 0 |
| ST258 | 106 (100.0) | 44 (41.5) | 2 (1.9) | 2 (1.9) |
| ST307 | 68 (100.0) | 22 (32.4) | 1 (1.5) | 0 |

ST: sequence type
